# 11 A Multi-Pronged Educational Approach to Heighten Nurse Confidence with Burn Fluid Resuscitation

**DOI:** 10.1093/jbcr/iraf019.011

**Published:** 2025-04-01

**Authors:** Mark Romero, Stacey Richerbach, Tiffany Hockenberry, Jennifer Granger, Cheryl Barela, Karen Richey, Kevin Foster

**Affiliations:** Diane & Bruce Halle Arizona Burn Center; Diane & Bruce Halle Arizona Burn Center; Diane & Bruce Halle Arizona Burn Center; Diane & Bruce Halle Arizona Burn Center; Valleywise Health Medical Center; Diane & Bruce Halle Arizona Burn Center; Diane & Bruce Halle Arizona Burn Center

## Abstract

**Introduction:**

In 2023, our center implemented a novel clinical decision support system (CDSS) for burn resuscitation. Initial education was provided to staff and performance was monitored in real-time. It was found that burn nursing staff (RN) were struggling with the components of CDSS documentation despite ongoing education. Recognizing that RNs lacked understanding, confidence and comfort with the system, the team revamped the CDSS education. A quality improvement (QI) project was initiated using a multi-pronged educational approach. The purpose of this study was to evaluate the efficacy of the project in improving nurse-reported levels of comfort and confidence.

**Methods:**

The Burn Educator and Burn Nurse Specialist worked to expand the existing education mediums which included nursing huddles and instructional handouts. Complementary resources were developed, namely classroom training, an enhanced step-by-step guide, and initiation checklist. Learners were given pre- and post-education surveys to ascertain comfort and confidence in CDSS initiation, ongoing management, troubleshooting, location of resources and serving as a resource for colleagues. A post-education test was administered with a minimum score of 100% required to pass.

**Results:**

A total of 33 RNs completed the training. The greatest improvement in comfort and confidence was seen in the management of resuscitation in the operating room (pre 30.3%, post 78.8%), followed by initiation and troubleshooting (pre 48.5%, post 93.9%), supporting colleagues with CDSS use (pre 48.5%, post 72.7%), and ability locating resources (pre 69.7%, post 97%). Ultimately, all RNs passed the post-test with the required 100% score.

**Conclusions:**

The most notable improvements were seen in comfort initiating CDSS-guided fluid resuscitation and continuing resuscitation off-unit. These measures of amplified comfort and confidence, combined with increased levels of understanding ultimately impact nursing competency. All burn ICU and ED RNs are required to complete and pass the education prior to caring for a patient during fluid resuscitation in our center. The education is required annually and routine monitoring of performance is incorporated into our formal QI program.

**Applicability of Research to Practice:**

This project highlights the importance of expanding education to mediate fluctuating levels of RN comfort, confidence and understanding.

**Funding for the Study:**

N/A

